# Ability of a Blue Hemoglobin-Based Liquid as a Novel Technology to Stain Initial Enamel Demineralization: A Proof-of-Concept in vitro Study

**DOI:** 10.1159/000528413

**Published:** 2022-11-30

**Authors:** Anahita Jablonski-Momeni, Mara Müller, Heike Korbmacher-Steiner, Peter Bottenberg

**Affiliations:** ^a^Department of Orthodontics, Philipps-University Marburg, Medical Faculty, Dental School, Marburg, Germany; ^b^Department of Oral Health Care, Université Libre de Bruxelles (ULB), Brussels, Belgium

**Keywords:** Enamel caries, Artificial lesion, Smooth surface, Orthodontic brackets, BlueCheck

## Abstract

During orthodontic treatment, enamel demineralization can occur. Its early detection is the basis for efficient preventive measures to arrest or remineralize lesions. In the present study, the application of a novel blue hemoglobin-based liquid (BlueCheck) was evaluated as proof of concept for detection of artificially demineralized smooth surfaces. 60 samples from extracted human posterior teeth were randomly assigned to four groups (15 per group). In 30 of these samples (groups A and B), superficial enamel was removed to create a ground surface. On the surface of other 30 samples (group C and D), orthodontic metal brackets were bonded. On each surface, BC liquid was applied and rinsed with water after 3 min (baseline). All surfaces were checked by two independent observers for presence of blue areas. On each sample, one side was covered by nail varnish to protect this enamel part from demineralization. The samples were demineralized with lactic acid (pH 4.6) for 7 days (group A and C) and 14 days (group B and D), respectively. Mineral loss was determined using quantitative light-induced fluorescence after demineralization. BlueCheck dye was again applied on the samples and evaluated for presence of stained areas. Histological sections were prepared from randomly selected samples and lesion depth was measured. Kruskal-Wallis test was used for group comparison (α = 0.05). After demineralization, median ΔF value for all samples was −8.25% indicating the presence of an initial demineralization. The difference of ΔF values was not statistically significant between samples at 7 or 14 days of demineralization, nor for samples with and without orthodontic brackets (*p* = 0.13). At baseline, none of the sample surfaces showed discoloration, whereas a distinctive blue color was visible after demineralization in all samples exposed to acid-exposed areas, corresponding to 100% sensitivity. The internal control surfaces (without demineralization) did not show any staining, corresponding to 100% specificity. Histologically measured lesion depths ranged between 200 and 254 μm. In this in vitro study, staining of demineralized enamel surface areas were shown to be reliable. Based on our results, this easily applicable product seems useful to be an adjuvant method to clinical examination to monitor oral health during an orthodontic treatment on tooth surfaces after removal of dental biofilm.

## Introduction

Patients undergoing orthodontic treatment with fixed appliances can be regarded as a specific risk group. Next to the usual sites at risk like occlusal fissures or approximal surfaces, initial enamel demineralization can be found on smooth surfaces. Fixed orthodontic appliances form new sites for biofilm development and retention on surfaces otherwise easily accessible for oral hygiene measures. Pathogenic biofilms may cause enamel demineralization as well as inflammation of the gingiva [Loe et al., 1965]. Typically, demineralization is visible as a circular zone round the bracket attachment continuing in the direction of the gingival crevice and possibly other areas covered by the appliance [Bishara and Ostby, 2008].

In a meta-analysis, Sundararaj et al. [2015] found an incidence of 49% and a prevalence of 68% for initial carious lesions in patients undergoing fixed multibracket orthodontic treatment. Ren et al. [2014] reported that 60% of patients undergoing orthodontic treatment developed complications due to biofilm presence such as demineralization or gingivitis.

Prevention of initial enamel lesions should be an important objective of orthodontic practitioners and dentists. It is the responsibility of orthodontists to prevent enamel demineralization and plaque-induced gingivitis by choosing a suitable prophylactic system [Kirschneck et al., 2016]. The incidence and prevalence of enamel demineralization and plaque-induced gingivitis are closely related with the willingness of patients to use sufficient preventive measures [Julien et al., 2013]. Preventive measures need to be coordinated in close cooperation between orthodontists and referring dentists, who often support the prophylactic management of patients, albeit to different degrees [Helminen and Vehkalahti, 2003].

In a study by Øgaard et al. [1988], specially designed orthodontic bands for accumulation of dental biofilm were attached to premolars scheduled to be extracted as part of an orthodontic treatment. Active noncavitated lesions were seen within 4 weeks in the absence of biofilm removal and fluoride. The authors concluded as a clinical significance of the study that enamel demineralization associated with fixed orthodontic therapy is an extremely rapid process caused by a high and continuous cariogenic challenge in the plaque developed around brackets and underneath ill-fitting bands. Consequently, careful inspection of the appliance at every visit and preventive fluoride programs are therefore required [Øgaard et al., 1988].

On the long run, these lesions present as well a health as an aesthetic problem. Visually, enamel surface alterations can be detected up to 5 years after bracket removal [Øgaard, 1989]. With the growing demand for fixed orthodontic treatment, an increase of prevalence of biofilm-associated diseases can be expected.

In general, the caries assessment is based on a visual examination of clean teeth. Detecting smaller initial stage caries lesions may be more difficult as they develop in areas of plaque stagnation; moreover, during orthodontic treatment with fixed appliances initial lesions are not always easily detectable if dental biofilm or even gingival hyperplasia are present. Thus, removing plaque is essential before clinical examination [Martignon et al., 2019]. In cases of gingival hyperplasia, its remission should be promoted by adequate management of the dental biofilm such as mechanical or combination of mechanical and chemical control [Chesterman et al., 2017]. Subsequently, an appropriate clinical examination for caries should be performed.

However, visual and tactile caries detection is often subjective and adjunct methods can provide additional benefit in objectifying and documenting demineralization [Jablonski-Momeni et al., 2021]. Various methods for the operator-independent detection of initial demineralization such as fluorescence or bioluminescence-based techniques have been evaluated recently [Guerrieri et al., 2012; Gimenez et al., 2021; Jablonski-Momeni et al., 2022]. However, at present none of these techniques is frequently used in daily clinical practice. For a widespread application of such techniques next to high sensitivity and specificity, easy handling and time as well as cost efficiency are of prime importance [Gomez, 2015].

A new patented technique named BlueCheck (BC) has recently been developed for detection of initial carious lesions via reversible staining of porous bioapatite (Incisive Technologies Pty Ltd., Melbourne, Australia: kit and method for detecting porous dental hydroxyapatite, European Patent No. 2 547 311 B1, https://patents.google.com/patent/EP2547311B1/en). Stemming from work on molar incisor hypomineralization [Mangum et al., 2010], which demonstrated that hydroxyapatite-binding proteins are excluded from enamel porosities that have an intact surface layer, this new technology utilizes the natural hydroxyapatite-binding function of hemoglobin to specifically target a dark blue dye to regions of mineral porosity. BC can bind specifically to porous hydroxyapatite rapidly (t_1/2_ < 60 s), enabling sensitive differentiation between carious and sound enamel. Because BC's mechanism of action is direct binding to mineral crystals exposed by demineralization, the clinical readout allows direct visualization of the location of demineralization on the tooth surface with a high-contrast blue color, without the need for ancillary equipment. BC bound to regions of mineral porosity can be removed with sodium-lauryl sulfate containing toothpaste.

BC is an engineered biomolecule that consists of a deep-blue dye (amido black) covalently linked to a protein (hemoglobin) that has an affinity for porous hydroxyapatite (European Patent No. 2 547 311 B1). BC utilizes the natural hydroxyapatite-binding chemistry of proteins to specifically and reversibly bind to porous dental hydroxyapatite [Mangum et al., 2010]. The binding process is governed by an electrostatic reaction between the protein and the porous enamel [Fargues et al., 1998], allowing the stain to be removed easily using normal toothpaste containing a detergent. The liquid is painted onto the teeth and blue regions are identified showing the location of demineralization with a high-contrast blue color (European Patent No. 2 547 311 B1).

At present, no studies are published about this new detection aid. This proof-of-concept study aimed to evaluate the application of BC indicator (hereinafter referred to as BC liquid) for the detection of artificial demineralization on smooth surfaces with and without brackets in vitro.

## Materials and Methods

### Sample Selection and Sample Size Calculation

The study was performed on human extracted permanent molar teeth. The use of extracted teeth was approved by the Ethics Committee of the Medical Faculty of the Philipps University of Marburg, Germany (approval number (AZ 132/19). Prior to extraction, written informed consent was obtained from each patient for the use of the extracted teeth for study purposes.

**Fig. 1:**
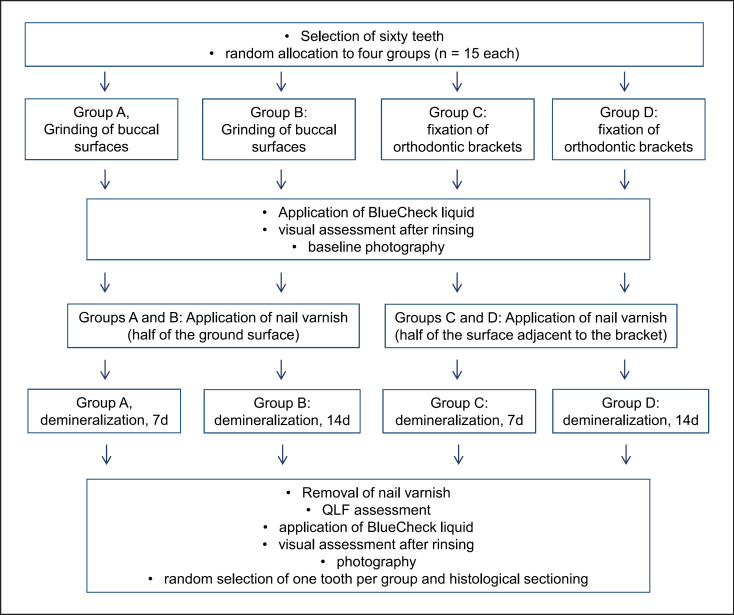
Flowchart representing the study design. QLF, quantitative light-induced fluorescence.

The majority of the teeth were impacted third molars; however, molar or premolar teeth extracted due to periodontal disease or other reasons were also collected and examined for inclusion. The teeth were extracted in a dental office by dentists not involved in the study. The indication for extraction was taken independently of the investigators by the treating dentist and teeth were collected at a later time for laboratory use. The research did not involve human participants. All methods were carried out in accordance with relevant guidelines and regulations. A flowchart with the study design is displayed as Figure 1.

After extraction, teeth were stored in a 0.9% NaCl solution containing 0.001% sodium azide at 6°C. Before selection, teeth underwent cleaning with scalers and a rotating bristle (Pluradent GmbH, Offenbach, Germany) and polishing paste (Clinpro Prophy Paste, 3M ESPE, Seefeld, Germany). The remaining paste was removed with a multifunctional syringe using water and air, and the teeth were stored in distilled water afterward. Inclusion criteria were teeth presenting a sound enamel layer; exclusion criteria were the presence of carious lesions, restorations, discolorations, hypomineralized areas or mineralization disorders. The selection of the teeth was performed after air-drying and observation under a microscope at 10-fold magnification (Leica Z6 APO, Wetzlar, Germany). The crowns were hemisected in mesio-distal direction using a diamond-coated band saw (EXAKT 300/310 CP; EXAKT Advanced Technologies GmbH, Norderstedt, Germany).

Sample size calculation was performed with MedCalc for Windows, version 20.010 (MedCalc Software, Ostend, Belgium, www.medcalc.org), based on a pilot study. A number of 14 samples were calculated (power 90%, α = 0.05), assuming detection of >85% of demineralized surfaces. In order to compensate for possible sample loss, 15 samples per group were chosen.

### Sample Preparation

Sixty samples of smooth surfaces were randomly assigned to 4 groups (*n* = 15 per group). The center of 30 samples (groups A and B) were grounded on waterproof silicon carbide paper (1,200 and 2,400 grit). On the surface of other 30 samples (group C and D), orthodontic metal brackets (discovery smart, Dentaurum, Germany) were bonded following the instructions for use, already described in other published studies [Jablonski-Momeni et al., 2021]. In brief, the enamel surface was etched and rinsed with water. The teeth were dried and orthodontic brackets were bonded in the center of each sample (Transbond XT primer and adhesive; 3M Unitek, Landsberg, Germany) and were light cured for 10 s (FlashMax P4 Ortho Pro, orthodontic light pen; CMS Dental, Copenhagen, Denmark, Peak Output Intensity: 6,000 mW/cm^2^). All specimens were glued with the cut dentine faces of the samples on plexiglass slides (25 mm × 75 mm × 2 mm, EXAKT Advanced Technologies GmbH, Norderstedt, Germany) and stored in distilled water at 6°C until further use.

### Baseline Measurements with BC Liquid

As baseline measurements, all samples were documented photographically using a dental camera (dentaleyepad, doctorseyes GmbH, Ochsenhausen, Germany) and a microscope at 10-fold magnification (Leica Z6 APO and QWin Standard V 3.4.0 Software; Leica Microsystems, Wetzlar, Germany). Then the BC liquid was applied using a microbrush and left undisturbed for 3 min according to manufacturer's instructions, followed by a rinse with water and air-drying the surface. Thereafter, all surfaces were checked for presence of blue areas under the microscope and new photographs were taken. Afterward, the samples were cleaned in an ultrasound bath containing 10% sodium lauryl sulfate as to manufacturer's instructions and a second BC liquid application was performed for reproducibility of the findings.

### Demineralization of the Samples and Fluorescence Measurements

On each sample, the right side was covered by an acid-resistant clear nail polish (ultra quick dry top coat, trend it up, dm, Karlsruhe, Germany) to protect this enamel part from demineralization and to create an internal control. The process of demineralization was performed according to established protocols [ten Cate et al., 1996] and is already described in detail elsewhere [Jablonski-Momeni et al., 2020]. In brief, the samples were demineralized in lactic acid (pH 4.6) buffered by a methylcellulose lactic acid gel. The samples were demineralized with lactic acid (pH 4.6, covered with a layer of an 8% methylcellulose) for 7 days (group A and C) and for 14 days (group B and D), respectively. During demineralization procedure, the samples were stored at 37°C (incubator type B; Heraeus GmbH, Hanau, Germany). After demineralization, the samples were removed from the gel, rinsed and cleaned with water jet from a three-in-one syringe to remove any gel remnants from the lesion. The nail varnish was removed from the surface. Then, the samples were air dried for further measurements and were photographed anew at 1- and 10-fold magnification. Fluorescence behavior (fluorescence loss, ΔF and ΔFmax) of the surfaces was determined using quantitative light-induced fluorescence (QLF) as a reference standard (Qraycam Pro, Inspektor Research Systems B.V.).

### Measurements with BC Liquid after Demineralization

Again, BC liquid was applied for 3 min followed by rinsing and drying. The surfaces were re-examined by the same observers for presence or absence of discoloration and photographs were taken to document color changes. After cleaning of the surfaces in an ultrasound bath containing 10% sodium lauryl sulfate, dye application was repeated.

### Histological Sections

From each group, one randomly selected sample was prepared for histological evaluation of lesion depth. Samples were dehydrated in an alcohol series, followed by infiltration with a mixture of PMMA resin (Technovit 7200 VLC; Kulzer GmbH, Hanau, Germany) and ethanol 70% (Fischar; Otto Fischar GmbH und Co. KG, Saarbrücken, Germany) for 24 h. Thereafter, samples were infiltrated with pure resin for 36 h and light polymerized in a dedicated appliance (Histolux, Exakt; Kulzer GmbH, Hanau, Germany). 650 µm sections were prepared after fixation of the samples to a plexiglass slide using the diamond-coated band saw, followed by grinding down to 100–150 µm with 2,500 and 4,000 grit waterproof silicon carbide paper. Microscopic photographs at 10- and 25-fold magnifications were obtained from the sections.

### Data Processing and Statistical Evaluation

After staining with BC liquid, samples were observed by two independent observers (A.J.M., M.M.) using naked eye and 10-fold magnified microphotographs. In each surface, the presence or absence of a discolored area was documented by a yes/no decision, without further quantification or classification of the discolored tooth surfaces.

QLF images were evaluated by the proprietary software (C4 Research software, v. 1.08). Per sample, the demineralized surface was marked and outcome variables ΔF and ΔFmax were analyzed. ΔF (%) corresponds to the average fluorescence loss in a surface and is related to the loss of mineral content and to lesion depth, while ΔFmax displays the highest value of ΔF measured within the region of interest and is an indication for the maximum lesion depth [Heukamp et al., 2022].

Histological images were analyzed using image-J software (ImageJ-win64.exe, version 1.46, National Institutes of Health, USA). Superficial and subsurface demineralization was observed as chalky-white or gray color change at ×10 magnification, depth was determined at 5 measuring points, and mean depth was calculated.

Statistical analysis was performed using MedCalc software. Normal distribution of fluorescence data was assayed with the Shapiro-Wilk test (*p* < 0.01) and further evaluation was done using nonparametric procedures (Kruskal-Wallis test) for group comparison. The level of significance was set at α = 0.05. The results of the BC liquid findings were represented using cross-tabulation.

## Results

**Fig. 2:**
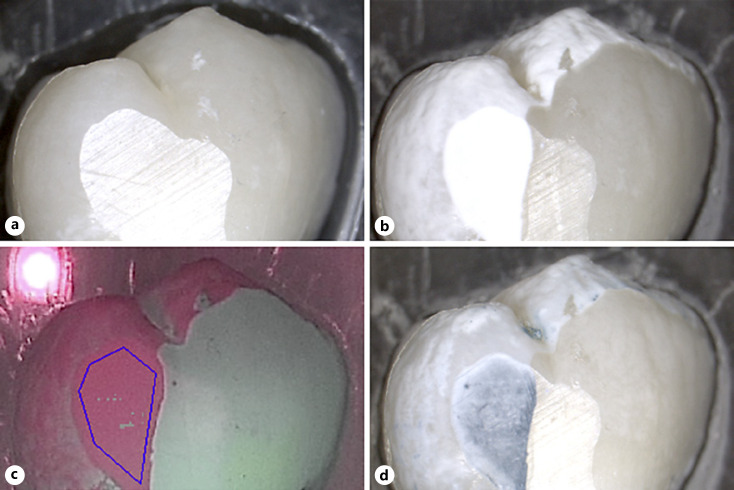
Image of a sample in group A (#09). **a** Image at ×10 magnification, prior to demineralization; **b** after demineralization; **c** corresponding QLF image (after demineralization). The blue line indicates the surface where the QLF measurement was performed: ΔF = −5.6%, ΔFmax = −5.9%; **d** after BC application.

**Fig. 3:**
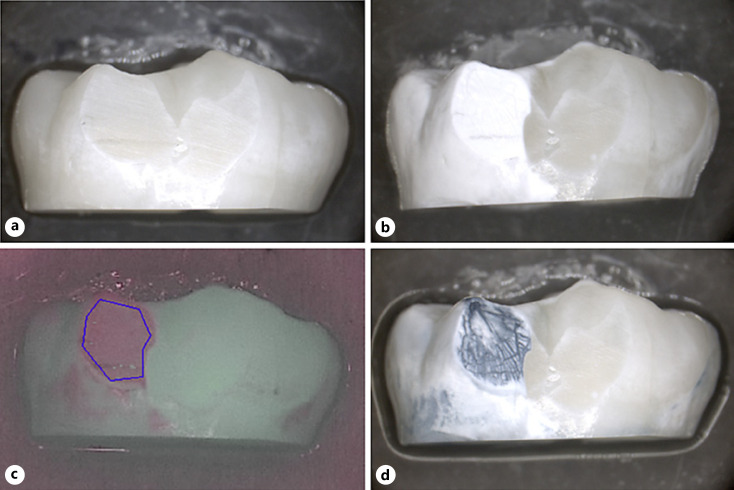
Image of a sample in group B (#04). **a** Image at ×10 magnification, prior to demineralization; **b** after demineralization; **c** corresponding QLF image (after demineralization). The blue line indicates the surface where the QLF measurement was performed: ΔF = −6.8%, ΔFmax = −11.0%; **d** after BC application.

**Fig. 4:**
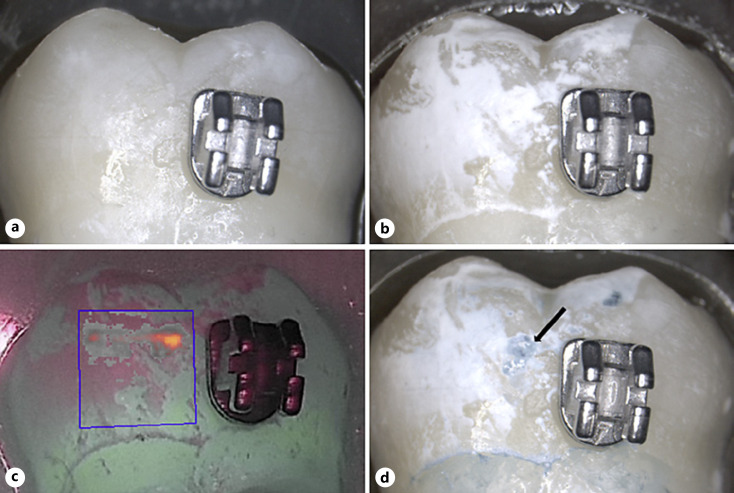
Image of a sample in group C (#05). **a** Image at ×10 magnification, prior to demineralization; **b** after demineralization; **c** corresponding QLF image (after demineralization) The blue line indicates the surface where the QLF measurement was performed: ΔF = −11.6%, ΔFmax = −28.7; **d** after BC application. The arrow indicates the area corresponding to ΔFmax.

All 60 samples could be evaluated. No BC liquid staining could be observed at baseline (Fig. 2, 3, 4, 5a) even after repeated application, corresponding to 100% repeatability on caries-free smooth surfaces.

After demineralization, all nail varnish-covered control surfaces remained unchanged, free of clinical signs of color and surface structural changes. The surfaces exposed to demineralization showed clear signs of chalky-white surface changes, clearly contrasting to the control side (Fig. 2, 3, 4, 5b). All exposed surfaces exhibited blue staining following BC liquid application while the unexposed control surfaces did not exhibit staining (Fig. 2, 3, 4, 5d). This corresponds to 100% sensitivity for the demineralized areas and 100% specificity for the unaffected smooth surfaces, whether there were orthodontic brackets or smooth surfaces only. The blue staining of the test surfaces could be reproduced after cleaning and repeated stain application in 100% of cases, corresponding to 100% repeatability of staining on demineralized smooth surfaces. The unexposed areas still remained without color change after the second staining.

After demineralization, all exposed surfaces showed a distinct fluorescence loss indicating the presence of initial lesions with a median ΔF of −8.25% (min −14.80%, max −5.30%) and a median ΔFmax of −17.60% (min −41.00%, max −5.4%), respectively. The results of the QLF measurements (average percentage of fluorescence loss relative to the fluorescence of sound tissue, ΔF, %) and maximum lesion depth (ΔFmax, %) are summarized in Tables 1, 2.

Inter-group comparison showed no significant differences between the fluorescence behavior of samples exposed to different demineralization times with or without brackets: *p* = 0.130 for ΔF and *p* = 0.243 for ΔFmax. QLF measurements of the unexposed enamel areas (covered by nail varnish) showed ΔF and ΔFmax values of 0 for each specimen, indicating a tooth surface without demineralization when compared to sound surrounding tissue (Fig. 2, 3, 4, 5c). Blue stain intensity was uniform in the flat ground lesions (e.g., surfaces without brackets), whereas in the lesions adjacent to orthodontic brackets the blue color was more inhomogeneous.

**Fig. 6:**
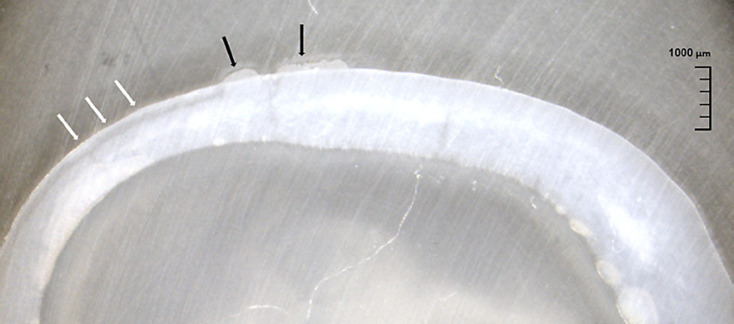
Histological section (sample C#13) at ×10 magnification. Demineralized area is on the left side of the sample (bright arrows). Sound enamel parts show a homogenous structure with a whitish color (right side of the sample) while demineralized areas are characterized by a lighter surface lesion and a darker appearing subsurface lesion. The remnants of the bracket adhesive are visible in the center of the section (dark arrows).

Histology showed in all evaluated samples a clear difference between sound and demineralized areas (Fig. 6). Mean depth of the surface lesion was 159.7 μm (105.2 μm–265.7 μm) adjacent to orthodontic brackets and 148.0 μm (116.9 μm–190.5 μm) in surfaces without brackets. All lesion depths are given in Table 3.

## Discussion

In the present study for the first time, a newly developed liquid caries indicator (BC) was tested for the use on artificial smooth surface enamel lesions which were free of dental biofilm (proof of concept). The dye was applied on tooth surfaces with or without brackets and free of dental biofilm according to a possible application in patients undergoing orthodontic treatment with fixed appliances. Measurements with established techniques, QLF and histology, show that stained surfaces mark areas with a pronounced mineral loss.

At present, several methods exist to support or facilitate caries detection [Gimenez et al., 2021; Janjic Rankovic et al., 2021; Kühnisch et al., 2022]. In daily clinical practice however, visual-tactile methods still are the standard, even if there is variability due to individual's standards and experience. Especially, the determination of lesion activity is normally based on visual or visual-tactile criteria [Drancourt et al., 2019], e.g., surface characteristics, such as change in texture, translucency, and color, and other factors, such as the presence of thick plaque and a plaque stagnation area, as well as gingivitis [Machiulskiene et al., 2020]. Implementation of preventive measures after early detection and activity assessment of demineralization, e.g., in orthodontic patients, can lead to lesion arrest and avoid restorative procedures.

It can be argued that a trained dentist would visually detect any demineralization. However, methods to visualize initial lesions can also be a means for patient communication and instruction in order to further acceptance and compliance. Besides, it was shown that patients' motivation plays a crucial and decisive role in maintaining favored oral hygiene and that it is worthy for orthodontists to put in additional efforts to motivate patients to maintain good oral hygiene [Huang et al., 2018].

A method for an objective and reproducible support of caries detection should be easily implemented in clinical procedures with a minimum of training; furthermore, cost efficiency is advantageous. Many available procedures and appliances such as optical methods (fluorescence–based cameras or devices) are connected with additional hard- and software acquisition, need technical know-how, and may be simply too onerous for practitioners.

As a caries detector dye, BC looks promising as it fulfills the requirements for clinical implementation. Existing staining methods are more suitable to detect dentinal caries and are intended as adjuvants in caries removal [McComb, 2000]. Presently available products such as CariesCheck (Nishika, Hikoshima, Japan), Caries Detector (Kuraray, Hattersheim, Germany), Caries Marker (Voco, Cuxhaven, Germany), or Snoop, the Caries Detective (Pulpdent, Le Thor, France), claim to facilitate minimally invasive caries removal. However, their purported mechanism is based on the presence of infected dentin [Javaheri et al., 2010]. As staining is positive for depolymerized collagen and not the presence of microorganisms, its specificity is not deemed to be sufficient [Boston and Graver, 1989]. Indicators for enamel demineralization however showing a reversible staining according to the requirements [Kumari et al., 2022] were not available until now.

Another approach has been the use of dyes to detect dental caries. However, these dyes are not selective for porous hydroxyapatite; they bind to proteins (presumed to be associated with infecting bacteria); or they occupy interstitial space, which reduces specificity and sensitivity. Moreover, these dyes cause the oral cavity to become discolored, bind to sound tooth surface, or require visualization with an irradiator [McComb, 2000].

Even if caries prevalence is decreasing in some countries, it is still a widespread disease. Polarization of prevalence and identification of risk groups remains a challenge for practitioners and public health authorities [Ziller et al., 2021]. Especially, children and adolescents during orthodontic treatment with fixed appliances present an elevated risk to develop enamel demineralization which can be undetected and without further preventive measures penetrate into dentin. This frequently observed side effect is in conflict with therapeutic goals, namely, improvement of the oral health situation. Therefore, an easy and objective detection of initial carious lesions is required to allow for a timely preventive or noninvasive approach.

Since the BC liquid is easy to use, this can also be a shortcoming when digital documentation is required. In order to store the results, taking photographs are necessary. Further limitation can be discussed which is the lack of a scale to quantify the color intensity of the stained areas. The decision about the presence or absence of a lesion is given as a yes/no answer, which is comparable to the visual assessment. Some attempts were made in the present study to quantify the color intensity of the sound and stained tooth areas, such as it was done in another study [Jablonski-Momeni et al., 2021]. There, luminescence spots which indicated active caries lesions were quantified by measurement of the pixels in the areas of interest. In the present study, it was not possible to perform quantification or automated analysis of the stained area with the available software. Natural tooth surfaces on themselves have a certain blue component. Human observers can, as shown in this study, detect the difference resulting in a very high sensitivity and specificity. Automated analysis must therefore, at least as far as our results show, proceed by human observers indicating stained areas for possible further data analysis. In the present study, grinding of the enamel surface led to a more homogenous demineralization in the samples without brackets rather than in surfaces with brackets which were not grounded. In the latter samples, the demineralized surfaces were stained blue, but with a lower intensity than in those areas with a deeper lesion. The most intense blue color in the surfaces was corresponding to the ΔFmax value in the area of interest (Fig. 4, 5d). However, no graduated classification of the blue color was done in the present study and methods should be developed in subsequent studies to quantify the intensity of the blue coloration. A categorical scale would be helpful for monitoring initial lesions, especially in case of active lesions where changes can normally be observed longitudinally to be sure that the initial assessment was valid [Pitts et al., 2021; Jablonski-Momeni et al., 2022].

In the present study, QLF was applied as reference standard for detection of demineralization. It is an established nondestructive method [Waller et al., 2012; Gomez, 2015; Heukamp et al., 2022] relying on a difference in light absorption and reflection between sound and demineralized dental hard tissues. The method shows a good correlation between mineral and fluorescence loss [de Josselin de Jong et al., 1995; Angmar-Månsson & ten Bosch, 2001; Waller et al., 2012]. QLF shows a high sensitivity and specificity for the detection of initial carious lesions on smooth surfaces [Park et al., 2019] and can be regarded as reference as well in in vivo and in vitro studies [de Josselin de Jong et al., 1995; van der Veen and de Josselin de Jong, 2000; Park et al., 2019; Heukamp et al., 2022] and adjacent to orthodontic brackets [Jablonski-Momeni et al., 2020].

Histological imaging in connection with digital image analysis still forms a gold standard in medical research and is regularly applied as reference for assessing the presence of demineralization [Wenzel and Hintze, 1999; Jablonski-Momeni et al., 2008]. In this study, it could be shown on selected samples that demineralization resulted in the typical artificial lesions with a light (surface) and dark (subsurface) zone [Silverstone 1967]. Lesion depths in this study are comparable to those reported in earlier studies [Featherstone and Rodgers, 1981].

### Conclusion and Clinical Relevance

Using BC liquid on surfaces which were free from dental biofilm, we were able to differentiate artificial lesions on smooth surfaces and adjacent to orthodontic brackets from sound enamel in a reliable and reproducible way. The product has not yet been evaluated in a clinical study and our results refer the in vitro proof of concept. Results from in vitro studies are not readily transferrable to the clinical situation; however, these findings are promising for further clinical research. At present, a quantification of blue stain is not feasible; detection is made based on a yes/no decision by a human observer. Based on our results, this easily applicable product seems useful to be an adjuvant method to clinical examination to monitor oral health during an orthodontic treatment on tooth surfaces after removal of dental biofilm. Furthermore, the method may be applied in clinical teaching; easy application and implementation in the student clinic makes it an interesting adjuvant tool in establishing a diagnosis and treatment planning. Possibly, BC liquid may also be applicable to monitor the effect of instated preventive measures to further remineralization such as application of fluoride or other agents.

## Statement of Ethics

The research was conducted ethically in accordance with the Word Medical Association Declaration of Helsinki. The study was performed on human-extracted permanent posterior teeth.

The use of extracted teeth was approved by the Ethics Committee of the Medical Faculty of the Philipps University of Marburg, Germany (approval number (AZ 132/19). Prior to extraction, written informed consent was obtained from each patient for the use of the extracted teeth for study purposes. The research did not involve human participants. All methods were carried out in accordance with relevant guidelines and regulations.

## Conflict of Interest Statement

The authors have no conflicts of interest to declare.

## Funding Sources

Except for the BC liquid and the orthodontic brackets, the project was funded by the authors' institutions. Incisive Technologies and Dentaurum had no influence on the design of the study, the data collection, or the presentation of the results.

## Author Contributions

Anahita Jablonski-Momeni, Mara Müller, Heike Korbmacher-Steiner, and Peter Bottenberg (all authors) designed the study and approved the final and revised version of this paper and are accountable for all aspects of this work. Anahita Jablonski-Momeni and Mara Müller performed the study.

## Data Availability Statement

All data generated or analyzed during this study are included in this article. Further inquiries can be directed to the corresponding author.

## Figures and Tables

**Fig. 5:**
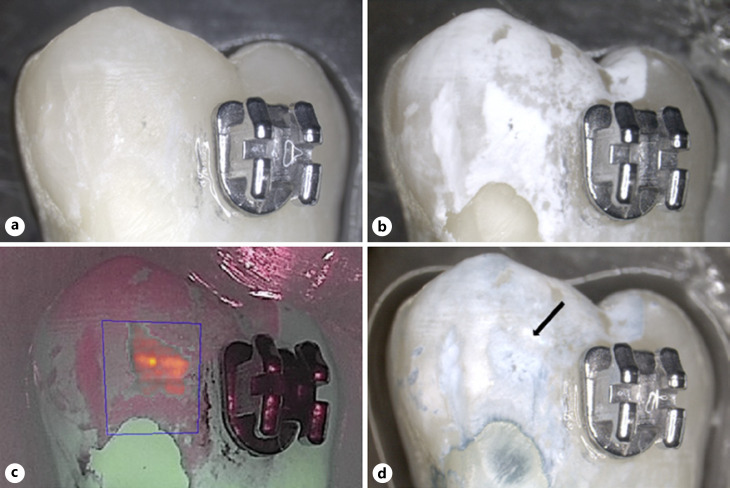
Image of a sample in group D (#14). **a** Image at ×10 magnification, prior to demineralization; **b** after demineralization; **c** corresponding QLF image (after demineralization). The blue line indicates the surface where the QLF measurement was performed: ΔF = −9.7%, ΔFmax = −38.6%; **d** after BC application. The arrow indicates the area corresponding to ΔFmax.

**Table 1:** Results of the QLF measurements in different groups after demineralization: ΔF (fluorescence loss [%]) shows average fluorescence loss in a lesion and is related to the loss of mineral content and to lesion depth (*n* = 15 per group)

Group	Minimum	Maximum	Median	Group comparison
All samples	−41.00	−5.40	−17.60	
A: without brackets, 7d demin	−37.10	−5.40	−14.60	*p* = 0.243
B: without brackets, 14d demin	−41.00	−10.00	−17.70	
C: with bracket, 7d demin	−28.70	−7.40	−21.20	
D: with bracket, 14d demin	−38.60	−9.50	−18.20	

**Table 2:** Results of the QLF measurements in different groups: ΔFmax (fluorescence loss [%]) shows the highest value of AF measured within the region of interest and is an indication for the maximum lesion depth (*n* = 15 per group)

Group	Minimum	Maximum	Median	Group comparison
All samples	−14.80	−5.30	−8.25	
A: without brackets, 7d demin	−14.50	−5.30	−7.10	*p* = 0.130
B: without brackets, 14d demin	−14.80	−6.30	−8.10	
C: with bracket, 7d demin	−12.70	−5.90	−9.00	
D: with bracket, 14d demin	−14.70	−6.60	−9.60	

**Table 3:** Mean values of lesion depth obtained by histology (in µm, based on 5 measurements per sample)

Group	Surface lesion	Subsurface lesion
A#09: without brackets, 7d demin	132.92	82.48
B#04: without brackets, 14d demin	163.21	52.85
C#13: with bracket, 7d demin	130.20	69.39
D#04: with bracket, 14d demin	189.20	64.22
